# Long-term late effects in older gastric cancer survivors: Survival analysis using Cox hazard regression model by retrospective electronic health records

**DOI:** 10.1007/s00520-023-08202-7

**Published:** 2023-12-15

**Authors:** Misun Jeon, Hyoeun Jang, Heejung Jeon, Chang Gi Park, Sanghee Kim

**Affiliations:** 1 College of Nursing and Brain Korea 21 FOUR Project, Yonsei University, Seoul, South Korea; 2 Department of Nursing, Graduate School, Yonsei University, Seoul, South Korea; 3 College of Nursing, University of Illinois, Chicago, IL USA; 4 College of Nursing and Mo-Im Kim Nursing Research Institute, Yonsei University, 50-1 Yonsei-Ro, Seodaemun-Gu, Seoul, 03722 South Korea; 5 Department of Artificial Intelligence, College of Computing, Yonsei University, Seoul, South Korea

**Keywords:** Older cancer survivor, Gastric cancer, Late effect, Survivorship care, Survival outcome

## Abstract

**Purpose:**

Because the population of older gastric cancer survivors (GCSs) is growing, understanding the long-term late effects experienced by these GCSs and their impact on survival outcomes is crucial for optimizing survivorship care. This study aims to identify and characterize these effects and investigate their association with survival outcomes.

**Methods:**

A retrospective analysis of electronic health records was conducted on 9,539 GCSs diagnosed between 2011 and 2017. The GCSs were divided into two age groups (< 65 and ≥ 65 years) and the long-term late effects were categorized by age using Cox proportional hazard models. The impact of clinical factors and age-specific late effects on survival was evaluated in the older GCSs.

**Results:**

Among the total GCSs, 37.6% were over and 62.4% were under 65 years of age. Significant differences between the age groups were observed in the cumulative hazard ratios (HRs) for iron and vitamin B12 levels and prognostic nutritional index (PNI) scores. In older GCSs, abnormal iron levels (HR 1.98, 95% CI 1.16–3.41, *p* = .013) and poor PNI scores (HR 1.59, 95% CI 1.03–2.47, *p* = .038) were associated with poorer survival outcomes. Additionally, being female was identified as a risk factor for lower survival rates (if male, HR 0.42, 95% CI 0.18–0.98, *p* = .045).

**Conclusion:**

This study highlights the typical long-term late effects experienced by older GCSs. By tailoring survivorship care to address nutritional-, age-, and gender-related factors, the overall survival and quality of life of older GCSs can be improved.

## Introduction

Gastric cancer is one of the most common types of cancer, ranking fifth in incidence and fourth in mortality worldwide. It was estimated that, by 2020, there would be more than one million new cases and 768,793 deaths from gastric cancer, accounting for 1 in 13 deaths worldwide [1]. Nevertheless, advancements in early detection methods, surgical techniques, and targeted therapies have contributed to the relatively lower mortality rates of gastric cancer compared to other cancers over the past decade [1]. In particular, the five-year relative survival rate for gastric cancer remains high at 33% as of 2023 [2]. As a result, the number of gastric cancer survivors (GCSs) has increased.

Older adults are the most vulnerable to gastric cancer, whose incidence and mortality rates increase progressively with age. Approximately 60% of new cases are diagnosed in older adults aged 65 years or older, with the average age of diagnosis being 68 years [3, 4]. Notably, 64% of cancer survivors are currently 65 years or older [5], and it is estimated that, by 2040, 73% of cancer survivors in the United States will be over the age of 65 [6].

Cancer survivors have reported an average of five symptoms that appear months or years after treatment [7]. These are called late effects—chronic conditions resulting from cancer treatment that cause physical and psychological problems, including secondary cancers [8]. Late effects are reported in all cancer types and commonly include a second primary cancer risk, anxiety, depression, trauma, cardiovascular disease risk, cognitive dysfunction, difficulties with employment and returning to work, pain, and sexual dysfunction [8]. In particular, GCSs have reported gastric cancer-specific late effects such as weight loss, diarrhea, chemotherapy-induced neuropathy, fatigue, poor bone health, indigestion, vitamin B12 deficiency, iron deficiency, postprandial fullness or eating dysfunction, dumping syndrome, and small intestinal bacterial overgrowth [9]. Cancer survivors experience these late effects five or more years after treatment ends, reporting a low quality of life (QOL) and high physical burden [7].

Addressing diverse late effects is crucial for formulating and implementing comprehensive care plans for cancer survivors. Several studies have been conducted on late effects in cancer survivors [10–12]. Specifically, older cancer survivors often experience a lower QOL than younger cancer survivors and older individuals without a history of cancer [13, 14]. However, studies on late effects in older GCSs, who account for a large proportion of cancer survivors, are scarce [15], with those examining the long-term late effects beyond five years in older GCSs being even rarer [15]. Therefore, this study analyzes electronic health records (EHRs) to determine the late effects of older GCSs and provide a fundamental understanding for optimal survivorship care management.

The research objectives include (1) identifying long-term late effects in GCSs; (2) describing the differences in long-term late effects by age group; (3) demonstrating the types of confirmed long-term late effects; and (4) exploring the related factor of overall survival in older GCSs. Based on this study’s findings, we hope to recommend appropriate interventions and future research directions, with the aim of improving the health-related QOL of older GCSs.

## Methods

### Study design and sample

This study employed a retrospective design utilizing clinical data extracted from EHRs at a Korean tertiary hospital. Participant eligibility criteria included having survived gastric cancer for at least five years, with a primary diagnosis of gastric cancer between 2011 and 2017, and having undergone either gastrectomy or endoscopic submucosal dissection (ESD). GCSs who were 65 years of age or older at the time of cancer diagnosis were categorized as older individuals [16]. Exclusion criteria included gastric cancer recurrence or death within five years of gastrectomy or ESD, and non-therapeutic gastrectomy (e.g., ESD for reasons other than gastric cancer or gastrectomy for the control of symptoms such as perforation, bleeding, or obstruction).

### Data collection

Demographic and clinical characteristics and late effects were extracted from EHRs for two specific time periods: the initial period of gastric cancer diagnosis and a subsequent period of five years or more post-diagnosis. The clinical characteristics compiled during the initial diagnosis period included age, smoking and drinking history, body mass index (BMI), cancer stage, and diagnostic details.

Long-term late effects were assessed based on the definition provided by the National Comprehensive Cancer Network (NCCN) [8, 9]. The collected data representing late effects are presented in Table 1. These late effects were recorded after five or more years following gastric cancer diagnosis. The recorded data included information on mortality, BMI, and laboratory results such as complete blood count and albumin levels, based on which the prognostic nutritional index (PNI) was calculated [17]. Other laboratory results such as vitamin B12, serum iron, serum vitamin D, and 25-OH-Vitamin D3 were also collected. Treatment history—including operations, ESD, chemotherapy, and radiotherapy—was documented. Bone mineral density and Mini-Mental State Examination (MMSE) results were also included in the analysis. The number of emergency department (ED) visits, which are not defined as late effects by the NCCN, were also analyzed to determine the healthcare management needs of GCSs five or more years post-diagnosis.

**Table 1:** Variables related to the late effects of the NCCN survivorship guidelines

Category	Late effect domain [8, 9]	Data collection in relation to the late effect
Late effects specific to gastric cancer survivors	Weight loss	BMI
	Diarrhea, indigestion, postprandial fullness, eating dysfunction	Prognostic nutritional index
	Bone health	Bone mineral density, vitamin D and 25-OH-Vitamin D level
	Vitamin B12 deficiency	Vitamin B12 level
	Iron deficiency	Serum iron
Common late effects in cancer survivors	Anxiety, depression, trauma, distress	Psychiatric consultation
	Cognitive function	MMSE
Late effects that are hard to classify	N/A	History of ED visits more than five years post-diagnosis

*BMI* body mass index, *MMSE* mini-mental state examination, *ED* emergency department

The PNI was calculated using the formula (10 × albumin level [g/dL]) + (0.005 × lymphocyte count [number/mm3]) [18]. GCSs were categorized into different groups based on their PNI. These groups included normal nutrition (PNI ≥ 50), moderate malnutrition (PNI = 40–49), severe malnutrition (PNI = 30–39), and serious malnutrition (PNI < 30) [18].

### Data analysis

Descriptive statistics were employed to examine the late effects of GCSs by age group. GCSs were divided into two groups: younger (aged < 65 years) and older GCSs (aged ≥ 65 years). Their demographic and clinical characteristics and the late effects were described. Comparisons of GCSs across age groups were performed using the t-test, Wilcoxon test, Cochran-Armitage trend test, Kruskal–Wallis rank sum test, and chi-square test. Statistical significance was determined using a two-sided p-value threshold of less than 0.05.

Kaplan–Meier cumulative hazard analyses and log-rank tests [19] were conducted on the two GCS groups to compare the cumulative risk for each of the following variables: history of ED visits (none versus 1 or more), history of MMSE tests (none versus 1 or more), iron level (normal versus abnormal, including deficient or high), and PNI (normal versus abnormal, including moderate, severe, or serious malnutrition). In this study, the event time was defined as the duration between gastric cancer diagnosis and the occurrence of late effects. A binary censoring variable was utilized to address censoring and indicate whether a late effect had been observed or not. The cumulative hazard ratio estimates the relative risk of experiencing the event between different age groups.

The Cox proportional hazard regression model was employed to estimate the hazard risk ratio and 95% confidence intervals for mortality, specifically within the older GCS group. The survival time was defined as the duration between gastric cancer diagnosis and death. To determine which variables were significant for survival (*p* < 0.05), each variable was evaluated in a separate univariate Cox regression analysis. To account for the joint effect on survival, we incorporated all factors that were significant in the univariate analysis, as well as variables for adjustment, into a multivariate model [19]. The Schoenfeld test was conducted as a proportional hazards assumption test. Data curation and analysis were performed using R software, version 4.1.0 [20].

## Results

### Demographic and clinical characteristics by age group

Of the 9,539 GCSs who underwent a gastrectomy or ESD between 2011 and 2017, 5951 (62.4%) and 3,588 (37.6%) belonged to the younger and older GCS groups, respectively (Table 2). Comparisons of the two age groups revealed that the proportion of GCSs with a history of drinking was significantly lower among the older group (χ ^2^ = 89.69, *p* < 0.001). Furthermore, there was a higher proportion of males in the older group (χ ^2^ = 26.13, *p* < 0.001), and the older group had a lower proportion of those diagnosed with a lower initial stage than did the younger group (χ ^2^ = 2.82, *p* = 0.004). However, the younger group had a higher proportion of those who underwent advanced treatment, such as gastrectomy or multimodal treatment, than did the older group (Z = 10.31, *p* < 0.001).

**Table 2:** The general and clinical characteristics of the study population

Category		Total *N* = 9539	Under 65 years *N* = 5951	Over 65 years *N* = 3588	t or χ ^2^ test or Cochrane–Armitage trend
		Mean ± SD or median (range) or *n* (%)	Mean ± SD or median (range) or *n* (%)	Mean ± SD or median (range) or *n* (%)	(*p*-value)
Age at diagnosis		59.89 ± 11.79	52.72 ± 8.45	71.78 ± 4.92	
All-cause mortality		856 (9.0)	439 (7.4)	417 (11.6)	48.87 (< .001^**^)
Median post-diagnosis follow-up for more than 5 years		2587 (1886–4215)	2618 (1886–4215)	2540 (1886–4187)	4.72 (< .001^**^)
Sex	Male	6328 (66.3)	3833 (64.4)	2495 (69.5)	26.13 (< .001^**^)
	Female	3211 (33.7)	2118 (35.6)	1093 (30.5)	
BMI at diagnosis	(kg/m^2^)	24.23 ± 26.88	24.21 ± 3.45	24.28 ± 17.34	
	Normal (18.5–22.9)	3674 (38.5)	405 (29.2)	1358 (37.8)	4.26 (.234)
	Underweight (< 18.5)	390 (4.1)	37 (2.7)	145 (4.0)	
	Overweight (23.0–24.9)	2517 (26.4)	411 (29.6)	990 (27.6)	
	Obese (≥ 25)	2931 (30.7)	516 (37.2)	1090 (30.4)	
	No information	27 (0.3)	19 (1.4)	8 (0.2)	
Smoking history	None	5295 (55.5)	3284 (55.2)	2011 (56.0)	1.21 (.546)
	Ex-smoker	3314 (34.7)	2071 (34.8)	1243 (34.6)	
	Current smoker	925 (9.7)	591 (9.9)	334 (9.3)	
	No information	5 (0.1)	5 (0.1)	0	
Drinking history	None	4522 (47.4)	2612 (43.9)	1910 (53.2)	89.36 (< .001^**^)
	Ex-drinker	3363 (35.3)	2183 (36.7)	1180 (32.9)	
	Current drinker	1642 (17.2)	1147 (19.3)	495 (13.8)	
	No information	12 (0.1)	9 (0.2)	3 (0.1)	
Initial stage	CIS	259 (2.7)	133 (2.2)	126 (3.5)	2.82 (.005^*^)
	I	6582 (69.0)	4097 (68.8)	2485 (69.2)	
	II	1019 (10.7)	642 (10.8)	377 (10.5)	
	III	1199 (12.6)	739 (12.4)	460 (12.8)	
	IV	249 (2.6)	186 (3.1)	63 (1.7)	
	No information	238 (2.5)	158 (2.7)	80 (2.2)	
Treatment type	ESD only	2432 (25.5)	1262 (21.2)	1170 (32.6)	152.60 (< .001^**^)
	Gastrectomy only	4382 (45.9)	2886 (48.5)	1496 (41.7)	41.42 (< .001^**^)
	Concurrent chemotherapy	2224 (23.3)	1538 (25.8)	686 (19.1)	26.13 (< .001^**^)
	Concurrent radiotherapy	276 (2.89)	205 (3.4)	71 (2.0)	16.60 (< .001^**^)

*SD* standard deviation, *BMI* body mass index, *CIS* carcinoma in situ, *ESD* endoscopic submucosal dissection

^*^p < .05; ^**^p < .001

Specifically, of the 249 stage IV GCSs who underwent ESD or gastrectomy, 228 (91.6%) received chemotherapy or radiation, and the remaining 21 (8.4%) received a combination of ESD and gastrectomy. Of all stage IV GCSs, 132 (53.0%) died. Of the stage IV GCSs, 63 (25.3%) were in the older group and 33 (52.4%) died.

### Characteristics of long-term late effects five years or more post-diagnosis by age group

Table 3 presents the differences in long-term late effects five years or more post-diagnosis between the two age groups. Significant differences in several long-term late effects—specifically vitamin B12 level (χ 2 = 4.021, *p* = .045), iron level (χ ^2^ = 104.62, *p* <.001), PNI score (t= 16.002, *p* <.001), and bone mineral density (χ ^2^ = 10.547, *p* <.001)—were observed between the age groups. No significant differences were found for other long-term late effects, such as the MMSE results (95% CI: 0.15-312.88, *p* = .511), recent BMI (χ ^2^ = 4.040, *p* = .257), and vitamin D level (t = 0.598, *p* = .550). There was a significant difference between the age groups regarding a history of ED visits more than five years post-diagnosis (W = 12138, *p* <.001). However, it should be noted that the number of screenings for cognitive function (*n* = 13) and bone mineral density (*n* = 23) were relatively small.

**Table 3:** Differences in long-term late effects between young and old adult groups five or more years post-diagnosis

Category (*n* = total/young age group/old age group)	Subcategory	Under 65 years (*N* = 5951)	Over 65 years (*N* = 3588)	t or Wilcoxon or Kruskal–Wallis rank sum or χ ^2^ test
		Mean ± SD or *n* (%)	Mean ± SD or *n* (%)	(*p*-value)
History of ED visits more than five years post-diagnosis (*n* = 358/187/171)	Once or more	187 (3.14)	171 (4.77)	0.72 (< .001^**^)
MMSE (*n* = 13/3/10)	Normal	2 (66.7)	3 (30.0)	4.09 (.511)
	Abnormal	1 (33.3)	7 (70.0)	
Vitamin B12 (*n* = 3248/2301/947)	(pg/mL)	485.37 ± 325.51	851.14 ± 639.68	4.02 (.045^*^)
	Normal	1813 (78.8)	692 (73.1)	
	Deficient or high	488 (21.2)	255 (26.9)	
Iron (*n* = 3088/2218/870)	(ug/dL)	121.78 ± 47.13	100.52 ± 35.57	104.62 (< .001^**^)
	Normal	666 (30.0)	409 (47.0)	
	Deficient	429 (19.3)	188 (21.6)	
	High	1123 (50.6)	273 (31.4)	
PNI (*n* = 4489/3027/1462)		54.45 ± 2.99	51.04 ± 3.82	16.00 (< .001^**^)
	Normal (≥ 50)	2594 (85.7)	981 (67.1)	
	Mild malnutrition (40–49)	334 (11.0)	362 (24.8)	
	Severe malnutrition (30–39)	65 (2.1)	80 (5.5)	
	Serious malnutrition (< 30)	34 (1.1)	39 (2.7)	
Bone mineral density (*n* = 23/15/8)	Normal	4 (26.7)	0	10.55 (< .001^**^)
	Osteopenia	9 (60.0)	1 (12.5)	
	Osteoporosis	2 (13.3)	7 (87.5)	
Recent BMI (*n* = 1598/1084/514)	(kg/m^2^)	22.06 ± 3.40	22.23 ± 3.17	4.04 (.257)
	Normal (18.5–22.9)	554 (51.1)	239 (46.5)	
	Underweight (< 18.5)	138 (12.7)	67 (13.0)	
	Overweight (23.0–24.9)	222 (20.7)	110 (21.4)	
	Obese (≥ 25)	170 (15.7)	98 (19.1)	
	UR	110 (60.1)	85 (67.7)	
Vitamin D (*n* = 646/476/170)	(ng/mL)	22.89 ± 13.12	21.98 ± 12.11	0.60 (.550)
	Normal	88 (18.5)	31 (18.2)	
	Deficient	73 (15.3)	29 (17.1)	
	Insufficient	314 (66.0)	110 (64.7)	
	High	1 (0.2)	0	

*SD* standard deviation, *BMI* body mass index, *ED* emergency department, *PNI* prognostic nutritional index

^*^p < .05; ^**^p < .001

### Cumulative risk of long-term late effects by age group

**Fig. 1:**
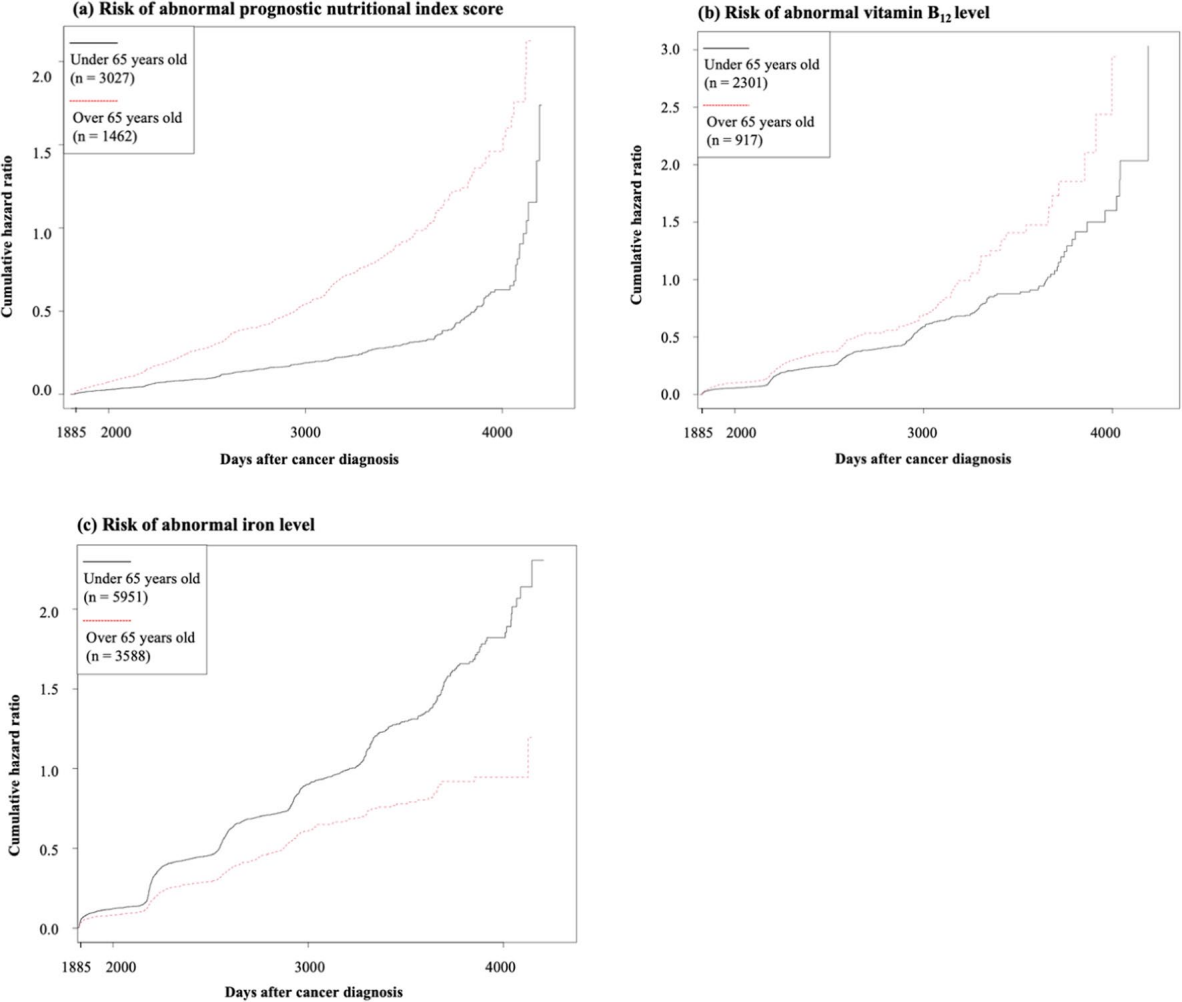
The cumulative hazard ratio of long-term late effects by age group five or more years post-diagnosis

Figure 1 demonstrates significant differences in the cumulative hazard ratio of long-term late effects between the age groups. Specifically, the cumulative hazard ratio of the iron level in the older group was 0.62 times (*p* < 0.001) lower than that of the younger group. Furthermore, the risk ratios of the PNI score and vitamin B12 level in the older group were 2.80 times (*p* < 0.001) and 1.42 times (*p* < 0.001) higher than those of the younger group, respectively.

### Cox proportional hazard ratio in older GCSs

**Fig. 2:**
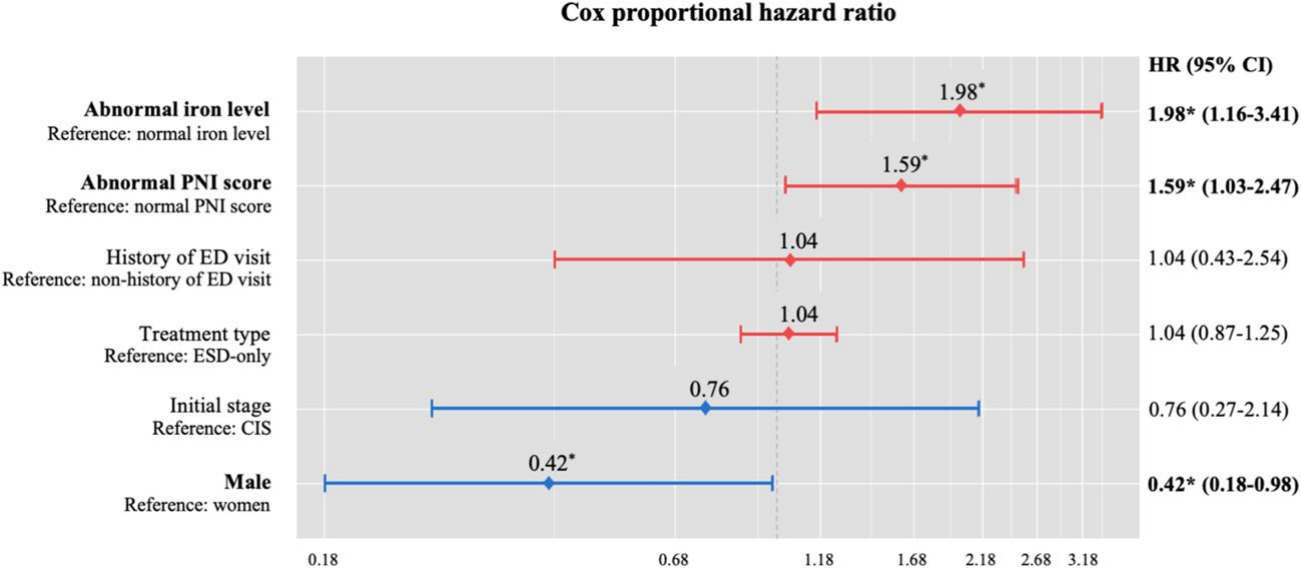
Cox proportional hazard ratios among older gastric cancer survivors five or more years post-diagnosis. ^*^p < .05; *PNI*: prognostic nutritional index; *HR:* hazard ratio; *CI*: confidence index; *ED*: emergency department; *CIS*: carcinoma in situ

In the univariate Cox regression analysis in which the variables significant for survival in older GCSs were selected, the PNI value (Hazard ratio [HR] 43.56; 95% confidence interval [CI] 5.92–320.36; *p* < 0.001), history of ED visits more than five years post-diagnosis (HR = 1.98; 95% CI 1.02–3.86; *p* = 0.045), and vitamin B12 level (HR = 3.87; 95% CI 1.42–10.51; *p* = 0.008) were significant. Figure 2 displays the Cox proportional hazard ratio for the survival of older GCSs. The Cox proportional hazard ratio of males in the older group was 0.42 times (95% CI 0.18–0.98;* p* = 0.045) lower than that of females. Additionally, the hazard ratios of abnormal iron results and PNI scores were 1.98 times (95% CI 1.16–3.41; *p* = 0.013) and 1.59 times (95% CI 1.03–2.47; *p* = 0.038) higher than those of normal iron results and PNI scores, respectively. However, the hazard ratios of the initial stage and treatment type did not show significant associations with the survival of older GCS individuals.

Additionally, in the younger group, the univariate analysis showed that the cancer stage at the time of diagnosis (HR = 2.82; 95% CI 1.93–4.10; *p* < 0.001), treatment type (HR = 6.70; 95% CI 2.64–17.00; *p* < 0.001), PNI value (HR = 90.71; 95% CI 12.27–670.63; *p* < 0.001), and history of ED visits more than five years post-diagnosis (HR = 1.76; 95% CI 1.02–3.03;* p* = 0.042) were significant predictors of survival.

## Discussion

This study demonstrated the long-term late effects experienced by GCSs who underwent a gastrectomy or ESD at a Korean tertiary hospital. It aimed to identify the risk factors associated with survival in older GCSs by analyzing EHRs. The findings revealed significant group differences in several long-term late effects, including the history of ED visits, vitamin B12 level, iron level, PNI score, and bone mineral density. The cumulative hazard ratios for abnormal iron levels, PNI scores, and vitamin B12 levels showed significant differences between the age groups. Furthermore, in the older group, the Cox proportional hazard factors associated with survival were an abnormal iron level, PNI score, and gender (specifically, being female). However, other late effects such as vitamin D level, BMI, and an abnormal MMSE result five years or more post-diagnosis did not reveal significant differences between the age groups.

Notably, according to the clinical characteristics observed in this study, older GCSs tend to be diagnosed with lower-stage cancer (72.7%). One might assume that this would result in high five-year survival rates among older GCSs. However, contrary to expectations, US cancer statistics reveal that individuals with gastric cancer over the age of 65 have the highest mortality rate among all age groups, accounting for 66.1% of deaths [21]. Notably, individuals over the age of 75 have a significantly lower survival rate of 25.2% [22]. These statistics demonstrate that older GCSs have lower chances of survival, even when diagnosed at an early stage. Older cancer survivors typically experience chronic disease [23], have poor functional status [24], and are at increased risk for adverse effects [25]. This may explain why older gastric cancer patients diagnosed at an early stage have lower survival rates. Therefore, when developing a cancer survivorship care plan for older gastric cancer patients, it is important to perform a geriatric assessment that goes beyond simply determining age or cancer staging to identify age-related conditions associated with poor treatment outcomes [26]. This highlights the need to establish age-differentiated survivor care protocols. By implementing an age-appropriate approach, healthcare providers can improve the overall care and outcomes of older GCSs.

This study found that older GCSs had poorer nutritional status than did younger GCSs. Digestive disturbances can contribute to nutritional imbalances [27]. In older adults, malnutrition leads to poor treatment outcomes and more side effects [28]. Poor nutritional status has been shown to impact survival in cancer survivors [27]. The National Cancer Institute has established diagnostic criteria for assessing nutritional status and managing nutrition-related issues, providing guidance for healthcare providers and patients [29]. Digestive symptoms experienced by cancer patients vary depending on the location of and treatment for cancer [29]. The findings emphasize the need to consider not only the nature of cancer—a traditional factor associated with nutritional status—but also age. BMI, one of the criteria reflecting nutritional status [30], was not identified as a significant variable affecting survival in this study. This may be due to the fact that BMI measurements were not routinely taken more than five years post-diagnosis. Recent research has examined indicators that may better reflect nutritional status [31], and PNI, which was a significant variable affecting survival in this study, may be an alternative. To achieve good nutritional status, regular nutritional screening using validated tools should be integrated into the survivorship plans for older GCSs. By adopting this approach, healthcare providers can effectively identify nutritional deficiencies and assess the impact of treatment and age on the nutritional status of older GCSs. Based on the results of this assessment, timely interventions can be implemented to address and improve nutritional deficiencies, even five years post-diagnosis. Implementing proactive nutrition management strategies can improve the overall survival and QOL of older GCSs.

In this study, older GCSs reported lower vitamin B12 levels than did younger GCSs. Vitamin B12 deficiency is a common late effect that occurs in 50% of individuals following gastrectomy [32]. Prolonged vitamin B12 deficiency can lead to various hematologic, neurological, and psychiatric disorders and increase the risk of cardiovascular disease [33]. Recent research has also identified an association between vitamin B12 deficiency and cognitive function, highlighting the importance of vitamin B12 interventions in older adults [34]. Aggressive vitamin B12 interventions are crucial from the start of treatment in older GCSs with low preoperative vitamin B12 levels, as they are more vulnerable to vitamin B12 deficiency after digital gastrectomy [35]. Older GCSs may require more intensive interventions to address vitamin B12 deficiency due to irreversible factors such as older age and having undergone a gastrectomy [32].

The study findings revealed that abnormal iron levels had different manifestations in younger and older GCSs. Younger GCSs were more likely to have iron overload, while older GCSs were more prone to have iron deficiency. Several factors contribute to iron-deficiency anemia, including poor nutritional status, which affects iron intake, and vitamin B deficiency, which results in poor iron absorption [36]. Older GCSs in this study had predisposing factors that contributed to iron-deficiency anemia, based on the fact that only 47% of older GCSs had normal iron levels, compared to 12% of those in community-living facilities [37]. This low percentage highlights the need for more extensive interventions beyond the current approaches recommended by the NCCN guidelines for addressing anemia after gastrectomy [9, 38]. The Global Burden of Disease (GBD) provides a standardized analysis of anemia thresholds, accounting for age, gender, cause, and region [39]. However, the clinical evidence available for use is limited to the World Health Organization’s [40] gender-specific thresholds (male: 13 g/dL or less, female: 12 g/dL or less) [40]. Considering this, we recommend proactive screening and intervention for groups with poor nutritional status or vitamin B12 deficiency—confirmed risk factors for iron-deficiency anemia—rather than focusing solely on isolated iron level measurements in clinical practice. Additionally, we suggest exploring cut-offs that consider age, gender, region, and comorbidity-specific characteristics to diagnose iron-deficiency anemia, thus incorporating the GBD’s findings into clinical application [39].

The cumulative hazard ratio analysis results indicate that abnormal iron levels, poor nutritional status (as indicated by PNI scores), and being female have a negative impact on the survival of older GCSs over a five-year follow-up period. These factors should be considered as predictors of survival in this specific population. This finding contrasts with that of a previous study that analyzed gastric cancer patients from 1992 to 2019, revealing that men had twice the mortality risk compared to women [41]. However, an analysis of time-to-diagnosis relative survival by race found that non-Hispanic Asian/Pacific Islanders male gastric cancer patients aged 65 and older had a lower five-year survival rate (20.1%) than females (15.4%) [22]. This difference is attributed to a lower incidence of local and regional staging in older women (57.2%) than in older men (60.6%) among non-Hispanic Asian/Pacific Islanders [22]. The gender disparities in outcomes have been explained by previous studies that have highlighted the role of intrinsic exposures or environmental risk factors, including female hormones [42, 43]. The existing cancer survivorship policies lack age and gender specificity, highlighting the need for tailored policies that reflect the characteristics of cancer survivors and influence treatment outcomes specific to the type of cancer. Predictive models utilizing big data can contribute to the refinement of these policies.

There are several limitations to this study. First, the retrospective design makes it potentially inaccurate when measuring all late effects. Assessment of late effects relies on tests performed when patients report symptoms or as recommended by follow-up protocols, which may not provide a comprehensive picture of all patients. In addition, the study only focused on long-term late effects of 5 years or more post-diagnosis, making it difficult to identify the correlation of late effects within 5 years. Conducting a prospective survey study to identify late effects in older GCSs could overcome this limitation and provide more comprehensive data. It may also be helpful to identify additional diagnoses or reasons for ED visits more than five years post-diagnosis to identify any late effects not defined by the NCCN, and to monitor symptoms seen in the older GCS population. Second, the generalizability of our findings is limited. This study was conducted in South Korea, where National Health Insurance coverage, which pays for cancer-related treatment, only extends up to five years post-diagnosis. Therefore, the transition from National Health Insurance to private health insurance may result in financial burdens related to medical payments, which may lead to fewer hospital visits. To address this limitation, future studies should consider using National Health Insurance data to examine late effects more comprehensively. Despite these limitations, this study's novelty lies in identifying not only how the long-term late effects experienced by older GCSs differ from those of younger GCSs, but also which long-term late effect factors affect survival in older GCSs.

## Conclusions

Increasing advancements in medical technology are anticipated to result in a larger population of older cancer survivors. Alongside the goal of improving treatment outcomes, recent healthcare objectives aim to support the successful reintegration of individuals into their daily lives after completing cancer treatment. This retrospective study focused specifically on older GCSs—a significant demographic—and aimed to identify the characteristics of long-term complications experienced by this population. By identifying these distinct factors, the study provides a foundation for the development of personalized survivorship care protocols that consider gender, nutritional status, and age. Furthermore, the findings can inform policy recommendations for targeted cancer survivorship care, ultimately leading to improved QOL for older GCSs.

## Data Availability

Please email Dr. Sanghee Kim (sangheekim@yuhs.ac) for more information.
